# Etanercept Ameliorates Cardiac Fibrosis in Rats with Diet-Induced Obesity

**DOI:** 10.3390/ph14040320

**Published:** 2021-04-01

**Authors:** Chia-Chen Hsu, Yingxiao Li, Chao-Tien Hsu, Juei-Tang Cheng, Mang-Hung Lin, Kai-Chun Cheng, Shang-Wen Chen

**Affiliations:** 1 Department of Exercise and Health Sciences, University of Taipei, Taipei City 110, Taiwan; eardoctorhsu@yahoo.com.tw; 2 Department of Otorhinolaryngology, Taipei City Hospital, Taipei City 110, Taiwan; 3 Graduate Institute of Gerontology and Health Care Management, Chang Gung University of Science and Technology, Guishan, Taoyuan City 613, Taiwan; manghung2468@gmail.com; 4 Department of Nursing, Tzu Chi University of Science and Technology, Hualien City 970, Taiwan; bebeli009@hotmail.com; 5 Department of Pathology, I-Shou University Medical Center, Yanchao District, Kaohsiung City 824, Taiwan; ed103797@edah.org.tw; 6 Department of Medical Research, Chi-Mei Medical Center, Tainan City 710, Taiwan; jtcheng5503@gmail.com; 7 Department of Pharmacy, College of Pharmacy, Tajen University, Pingtung 907, Taiwan; 8 Division of Cardiology, Chi-Mei Medical Center Liouying, Tainan City 736, Taiwan

**Keywords:** cardiac fibrosis, diet-induced obesity, etanercept, TNF-α inhibitor, rats

## Abstract

Diet-induced obesity (DIO) is considered the main risk factor for cardiovascular diseases. Increases in the plasma levels of tumor necrosis factor alpha (TNF-α) is associated with DIO. Etanercept, a TNF-α inhibitor, has been shown to alleviate cardiac hypertrophy. To investigate the effect of etanercept on cardiac fibrosis in DIO model, rats on high fat diet (HFD) were subdivided into two groups: the etanercept group and vehicle group. Cardiac injury was identified by classic methods, while fibrosis was characterized by histological analysis of the hearts. Etanercept treatment at 0.8 mg/kg/week twice weekly by subcutaneous injection effectively alleviates the cardiac fibrosis in HFD-fed rats. STAT3 activation seems to be induced in parallel with fibrosis-related gene expression in the hearts of HFD-fed rats. Decreased STAT3 activation plays a role in the etanercept-treated animals. Moreover, fibrosis-related genes are activated by palmitate in parallel with STAT3 activation in H9c2 cells. Etanercept may inhibit the effects of palmitate, but it is less effective than a direct inhibitor of STAT3. Direct inhibition of STAT3 activation by etanercept seems unlikely. Etanercept has the ability to ameliorate cardiac fibrosis through reduction of STAT3 activation after the inhibition of TNF-α and/or its receptor.

## 1. Introduction

Obesity is a growing public concern around the world. Obesity is considered the main risk factor for many diseases, including cardiovascular diseases, fatty liver, type II diabetes, and cancer [1]. Particularly, obesity is also linked to the current pandemic of coronavirus disease 2019 (COVID-19) [2]. Individuals with obesity showed significant increases in morbidity and mortality from COVID-19 [3]. In addition to genetic factors, the environmental parameters, including excessive food intake and a sedentary lifestyle, participate in the induction of obesity [4]. The Western-type diet, characterized by a high dietary intake of saturated fats and sucrose, induces atherosclerosis and hyperglycemia [5]. Additionally, chronic inflammation is linked to hyperlipidemia, which is also involved in the induction of cardiovascular diseases (CVD) [6]. The signs of inflammation that are associated with lipid accumulation and excess lipids may change the composition of cell membranes [7]. Therefore, inflammation due to a high fat diet (HFD) is responsible for metabolic syndrome, type-II diabetes, nonalcoholic fatty liver disease (NAFLD), and hepatic fibrosis, in addition to CVD. Similar changes were also observed in rats [8] and mice [9]. Based on the results of animal studies, HFD triggers acute and/or chronic inflammation via a complicated mechanism [10]. Chronic HFD has been demonstrated to induce cardiac hypertrophy and fibrosis directly in mice [11].

Increases in plasma levels of cytokines, including tumor necrosis factor alpha (TNF-α), interleukin-6 (IL-6), and interleukin-1 beta (IL-1β), are associated with inflammation [12]. Therefore, these cytokines are widely used as inflammatory markers in HFD-fed animals. Recently, cardiac inflammation has been described in animals receiving HFD [13]. The expression of TNF-α is higher in obese rats fed an HFD [14]. TNF-α is known to be involved in cardiac injury, acting via inflammatory pathways and/or activation of cell death programs including apoptosis [15]. Generally, TNF-α signaling is initiated through two receptors located on the cell-surface, namely, TNF receptor-1 (TNFR1) and TNF receptor-2 (TNFR2) [16]. TNFR1 and TNFR2 were shown to exert disparate and/or opposing effects during heart failure using TNFR1- and TNFR2-null mice [17].

Etanercept (Enbrel@), a recombinant human soluble tumor necrosis factor-α (TNF) receptor protein, is effective as a TNF-α inhibitor [18]. Etanercept can competitively block the binding of TNF-α with its receptor to reduce its activity [18]. In the clinic, etanercept is widely used to treat TNF-α-related diseases, including rheumatoid arthritis, psoriasis, ankylosing spondylitis, Crohn’s disease, diabetes mellitus, Alzheimer’s disease, and cancers [19]. Previous research indicated that TNF-α-antagonism attenuates the development of experimental diabetic cardiomyopathy associated with a reduction of intramyocardial inflammation and cardiac fibrosis [20]. In addition to heart failure, overexpression of TNF-α in mice promotes the occurrence of cardiac hypertrophy [21]. Therefore, etanercept has been shown to alleviate cardiac hypertrophy after inhibition of the TNF-α receptor [22].

In the present study, we investigated the effect of etanercept on cardiac fibrosis in diet-induced obese rats. Additionally, the potential mechanism(s) of the effects of etanercept in hearts were also evaluated.

## 2. Results

### 2.1. Effects of Etanercept on Changes in Body Weight, Blood Lipids Levels and other Parameters in HFD-Fed Rats

After the intake of an HFD, the body weight, and fasting blood glucose, in addition to total cholesterol, and triglyceride levels in the rats were significantly increased, compared with those in the normal control rats, as shown in Table 1. Etanercept markedly attenuated these values compared with vehicle-treatment (Table 1). Interestingly, the liver markers aspartate aminotransferase (AST) and alanine transaminase (ALT) were similarly changed (Table 1).

### 2.2. Effects of Etanercept on Cardiac Functions

**Figure 1:**
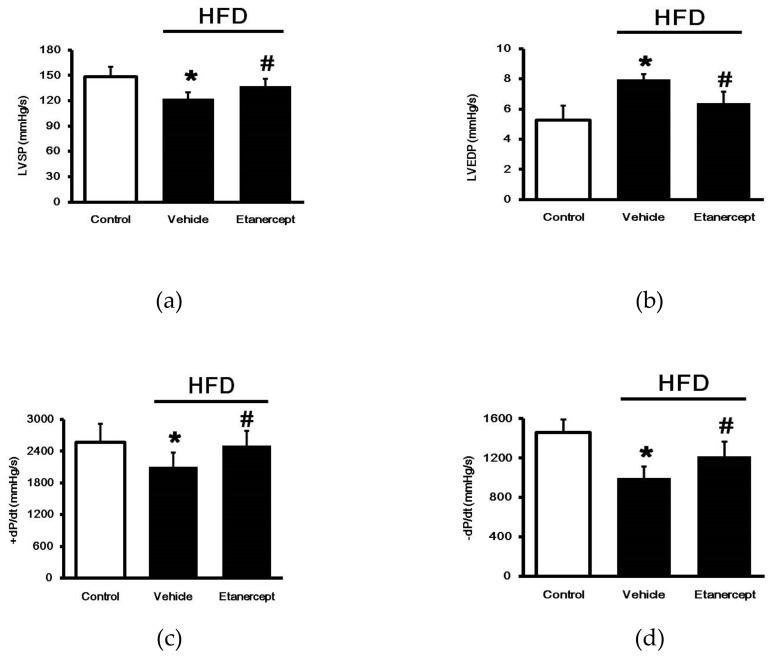
Effects of etanercept on cardiac performance in HFD-fed rats. Ventricular hemodynamic parameters in each group. (**a**) LVSP, (**b**) LVEDP, (**c**) +dp/dtmax and (**d**)-dp/dtmax. Treatment with etanercept reversed the changes in cardiac performance (+dP/dtmax and -dP/dtmax) in the HFD-fed rats. The data are shown as the mean ± standard deviation (*n* = 6). * *p* < 0.05 vs. normal control group; # *p* < 0.05 vs. vehicle-treated group, one-way ANOVA, followed by Bonferroni test. LVSP, left ventricular systolic pressure; LVEDP, left ventricular end diastolic pressure; +dp/dtmax, maximal rise rate of left ventricular pressure; -dp/dtmax, maximal fall rate of left ventricular pressure. HFD, high fat diet; LVSP, left ventricular systolic pressure; LVEDP, left ventricular end-diastolic pressure; dp/dtmax, the maximum rate of left ventricular pressure rises.

As shown in Figure 1, the HFD-fed rats showed a marked decrease in LV contractility including LVSP and max/min values of dp/dt, but an increase in LVEDP, compared to the rats fed a normal chow. However, the etanercept treatment in HFD-fed rats showed a significant recovery in LV contractility, compared to the vehicle-treated HFD-fed rats. Therefore, etanercept may alleviate HFD-induced damage to cardiac performance.

### 2.3. Effects of Etanercept on Cardiac Fibrosis in HFD-Fed Rats

**Figure 2:**
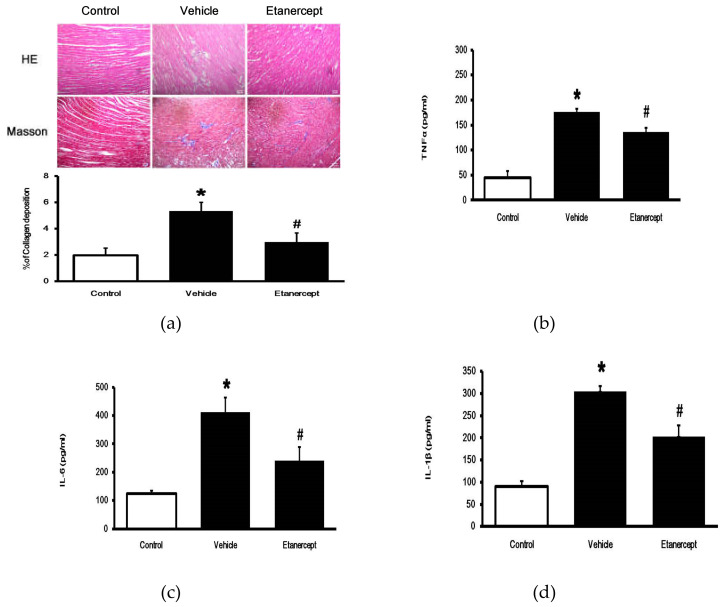
Effects of etanercept on heart histology and plasma cytokine levels of HFD-fed rats. Photomicrographs of the myocardium in the normal control rats (Control), the HFD-fed rats (Vehicle), and the etanercept treated HFD-fed rats (Etanercept) were characterized by staining with hematoxylin–eosin (HE Stain) and Masson’s trichrome (Masson Stain): histological section of the myocardium, the scale bar is 50 μM. Changes in collagen deposition are also compared (**a**). The plasma levels of TNFα (**b**), IL-6 (**c**), and IL-1β (**d**) were also compared among the three groups. The data are shown as the mean ± standard deviation (*n* = 6). * *p* < 0.05 vs. normal control group; # *p* < 0.05 vs. vehicle-treated group, one-way ANOVA, followed by Bonferroni test. HFD, high fat diet; TNFα, tumor necrosis factor-α; IL, Interleukin.

Cardiac fibrosis in HFD-fed rats was identified by pathological changes (Figure 2a) and higher levels of plasma cytokines. The plasma biomarkers, TNF-𝛼 (Figure 2b), IL-1β (Figure 2c), and IL-6 (Figure 2d), were significantly increased in blood of the HFD-fed rats compared with the normal control rats. After treatment, etanercept markedly alleviated the changes in these biomarkers. Additionally, Masson’s trichrome staining indicated greater fibrosis in the interstitial and perivascular regions of the myocardium in the HFD-fed group than in the control group. Moreover, HFD significantly increased collagen deposition in rat hearts (Figure 2a). The collagen deposition in the hearts of HFD-fed rats was substantially reduced by etanercept-treatment, compared to that in the vehicle-treated HFD-fed rats. Therefore, etanercept may ameliorate cardiac fibrosis induced by HFD in rats.

### 2.4. Effects of Etanercept on Fibrosis-Related Genes in the Hearts of HFD-Fed Rats

**Figure 3:**
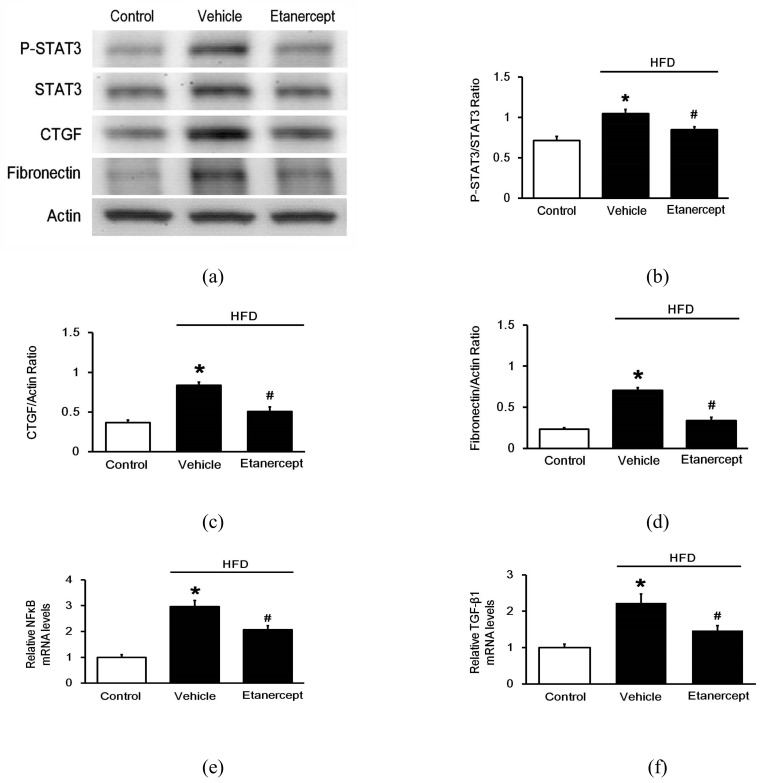
Effects of etanercept on fibrosis-related gene expression in the hearts of HFD-fed rats. Western blotting analysis of each protein in the myocardium from the normal control rats (Control), HFD-fed rats (Vehicle), and etanercept- treated HFD-fed rats (Etanercept) were compared; p-STAT3, STAT3, CTGF, and fibronectin were measured. Representative blots are presented (**a**); and the quantification of each signal, including STAT3 activation (p-STAT3/STAT3) (**b**), CTGF (**c**), and fibronectin (**d**) relative to the internal control was performed for comparison. The mRNA levels of NFkB (**e**) and TGF-β1 (**f**) were also measured using real-time qPCR. The results are presented as the mean ± standard deviation (*n* = 4 per group). * *p* < 0.05 compared with the control rats and # *p* < 0.05 compared with the vehicle treated rats, one-way ANOVA, followed by Bonferroni test. p-STAT3, phosphorylated signal transducer and activator of transcription 3; CTGF, connective tissue growth factor; NFkB, nuclear factor kappa-light-chain-enhancer of activated B cells; TGF-β1, transforming Growth Factor Beta1; qPCR, quantitative polymerase chain reaction.

Next, we investigated the levels fibrosis-related genes expression using Western blots to assess the protein levels (Figure 3a) and qPCR to examine the mRNA levels. In the hearts of the HFD-fed rats, fibrosis markers CTGF (CCN2) (Figure 3c) and fibronectin (Figure 3d) were both markedly elevated, compared to those in the hearts of normal rats. Interestingly, etanercept attenuated these changes in fibrosis biomarkers. Activation of STAT3 (p-STAT3/STAT3) in the hearts was similarly changed (Figure 3b). Additionally, the mRNA levels of NFkB (Figure 3e), a TNF receptor-regulated gene, and TGF-β1 (Figure 3f) were increased in the hearts of the HFD-fed rats. Treatment with etanercept also significantly reduced these changes.

### 2.5. Effects of Etanercept on Palmitate-Stimulated Fibrosis-Related Gene Expression in Cultured Cardio Myoblast H9c2 Cells

To understand the role of STAT3 activation in the effects of etanercept on cardiac injury, we used palmitate-stimulated H9c2 cells to mimic the animal model of HFD-fed rats, as described in a previous report [22]. It has been documented that palmitate significantly decreased cell viability in a time-dependent manner at doses less than 500 μM [23]. Upon sustained palmitate (100 μM) incubation for 24 h, TNF-ɑ and IL-6 secretion from H9c2 cells was markedly elevated [22]. Additionally, etanercept (50 μM) is known to ameliorate injury in H9c2 cells after a 48-h incubation [21]. Therefore, we used palmitate (250 μM) incubation for 48 h as the cell model and treated this model with etanercept (50 μM). Moreover, we applied a specific inhibitor of STAT3, Stattic [24], to investigate the role of STAT3 in H9c2 cells according to our previous report [25].

**Figure 4:**
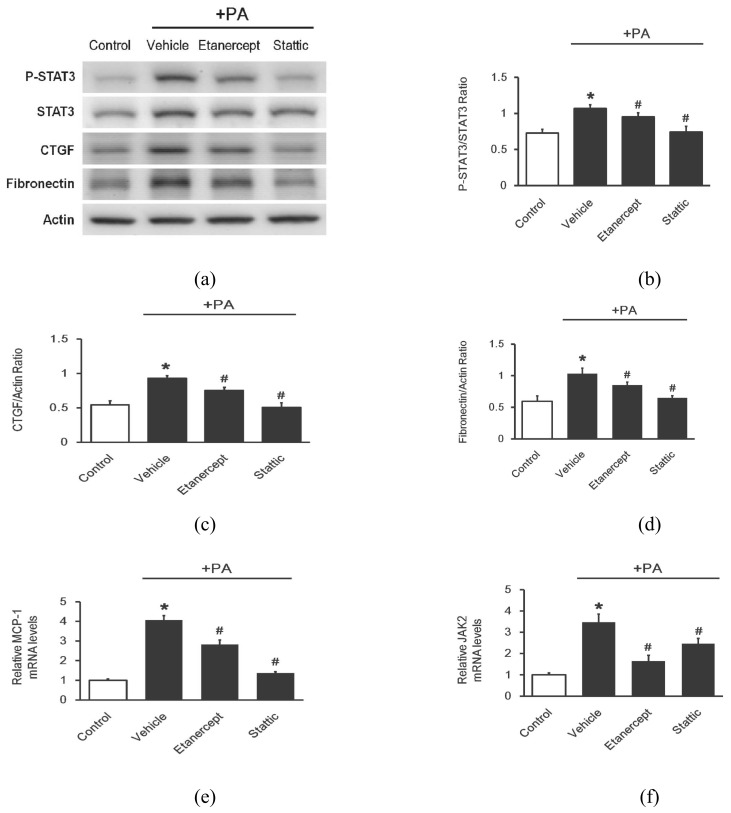
Effects of etanercept on the fibrosis-related gene expression in H9c2 cells stimulated with palmitate (PA). The cells incubated with palmitate (250 μM) for 48 h were used as the model to mimic the HFD-fed rats. Then, etanercept (50 μM) or Stattic (1 μM) was added at 1 h after palmitate stimulation. Western blots of each protein in the H9c2 cells including the non-treated control group (Control), the palmitate-stimulated cells shown as line including the group with vehicle (Vehicle), etanercept group (Etanercept), and Stattic group (Stattic), were compared; p-STAT3, STAT3, CTGF, and fibronectin were measured. Representative blots are presented (**a**); and the quantification of each signal including STAT3 activation (p-STAT3/STAT3) (**b**), CTGF (**c**), and fibronectin (**d**) relative to the internal control was performed for comparison. The mRNA levels of MCP-1 (**e**) and JAK2 (**f**) were also measured using real-time qPCR. The results are shown as the mean ± standard deviation (*n* = 4 per group). * *p* < 0.05 compared with the control group and # *p* < 0.05 compared with the vehicle-treated group, two-way ANOVA, followed by Bonferroni test. p-STAT3, phosphorylated signal transducer and activator of transcription 3; CTGF, connective tissue growth factor; MCP-1, monocyte chemoattractant protein-1; JAK2, Janus kinase 2; qPCR, quantitative polymerase chain reaction.

The protein levels of the fibrosis markers, CTGF (CCN2) (Figure 4c) and fibronectin (Figure 4d), were significantly elevated by palmitate. Treatment with etanercept inhibited both of these changes in a marked fashion. However, etanercept seemed to be less effective than Stattic (1 μM). Additionally, the activation of STAT3 (p-STAT3/STAT3) was also induced by palmitate (Figure 4b). Both etanercept and Stattic attenuated the action of palmitate, while Stattic showed a greater reduction than etanercept (Figure 4a). Similar changes were also observed in the mRNA levels of monocyte chemoattractant protein-1 (MCP-1): (Figure 4e).

Interestingly, the mRNA level of JAK2, which is the upstream of STAT3, was markedly elevated by palmitate, compared to the normal control (Figure 4f). This effect of palmitate was reduced by etanercept more significantly than by Stattic. The effects of cytokines binding with their receptors on JAK-STAT3 signal activation seems to be a possible explanation for this change in H9c2 cells.

## 3. Discussion

In the present study, cardiac fibrosis was induced in rats fed with HFD for 16 weeks as described previously [23,26]. STAT3 activation was associated with the increase in fibrosis-related factors, which is consistent with a recent report [27]. Notably, we found that etanercept is effective to ameliorate cardiac fibrosis in diet-induced obese rats through blockade of TNF-α to result in reduction of STAT3 activation. Cardiac protection by etanercept has been demonstrated in ischemic injury [28] and cardiac hypertrophy [22]. However, the effect of etanercept on cardiac fibrosis induced by HFD in rats has not been previously described.

High fat consumption is a major contributor to the development of insulin resistance, which can lead to type-2 diabetes [29]. The intake of a diet rich in lipids is associated with the development of cardiac damages [30]. High fat consumption is related to oxidative stress-dependent inflammatory injury [31]. The release of cytokines, which leads to the aggregation and infiltration of inflammatory cells, is a main step during inflammation [32]. HFD has been documented to induce TNF-α expression in tissues [14]. TNF-α secreted from macrophages may promote the inflammatory cascade by increasing the release of other cytokines and influencing the recruitment of neutrophils [33]. TNF-α showed a negative inotropic effect inhibiting myocardial contractility and lowering blood pressure [15]. Additionally, TNF-α can induce cardiac apoptosis and is involved in the ventricular remodeling [34]. Therefore, cardiac fibrosis in HFD-fed rats shown in the current study is consistent with the previous reports [23,26,35].

Etanercept is widely used as an effective TNF-α inhibitor [18], which competitively inhibits the binding of TNF-α to its receptor. Therefore, etanercept has been used to treat TNF-α related diseases in the clinic. TNF-α could also increase hepatic lipogenesis in obesity and diabetic animal model [36]. In the present study, we used etanercept at a dose effective for improving disordered behaviors in rats [37] and this dose is also the same as that used in mice [38]. We found that etanercept ameliorates the cardiac fibrosis induced by HFD in rats as assessed by biochemical indicators or pathological determinations. The etanercept treatment of rat reduced the HFD-induced body weight gain; and prevented elevations of serum total cholesterol levels, triglyceride levels, Low-density lipoprotein-cholesterol (LDL), ALT, and AST in HFD-fed rats. The high-density lipoprotein (HDL) cholesterol level was significantly increased after etanercept treatment. Etanercept treatment also normalized blood glucose levels in HFD rats. These results are consistent with previous research demonstrating that hyperglycemia was reduced in HFD animal treated with etanercept due to enhance glucagon-like peptide-1 secretion [39]. 

In addition to the changes in parameters similar to those in HFD-fed rats [40], etanercept also inhibits the activation of STAT3. Two common TNF-α inhibitors, etanercept and adalimumab, have been shown to decrease p-STAT3 in human Th17-polarized cells [41]. Evidence shows that STAT3 is an important regulator of cardiac fibrosis [42]. STAT3 mediates both inflammatory and noninflammatory responses downstream of cytokine activation, while initial STAT3 activation, which is linked to IL-6, is pro-inflammatory and can be harmful. Additionally, STAT3 accumulated in the nucleus also induces the expression of IL-6 and other proinflammatory genes [43].

We found that etanercept ameliorates cardiac fibrosis mainly due to a reduction in STAT3 activation. Therefore, the potential mechanism(s) were investigated in H9c2 rat cardiomyoblasts. According to a previously described method [44], palmitate was used to stimulate H9c2 cells. The changes observed in fibrosis-related gene expression were the same as those observed in HFD-fed rats and etanercept also markedly inhibited these changes. However, this action by etanercept was less effective than that by Stattic. Stattic, as a specific inhibitor of STAT3 [24], may inhibit fibrosis-related gene expression in H9c2 cells stimulated with palmitate. Therefore, a reduction in STAT3 activation seems to be important for the effects of etanercept on cardiac fibrosis induced by HFD in rats. Additionally, etanercept markedly inhibited the mRNA levels of JAK2 in H9c2 cells stimulated with palmitate, while Stattic treatment showed a smaller effect. It seems that changes in JAK2 are mainly associated with the IL-6 production and less related to STAT3 activation, as described previously [27].

Generally, TNF-α signaling is mainly induced through two receptors, TNF receptor-1 (TNFR1) and TNF receptor-2 (TNFR2) [16]. TNFR1 and TNFR2 have disparate and opposing effects on cardiac function during heart failure: TNFR1 exacerbates cardiac function and TNFR2 ameliorates cardiac function [17]. The interplay between TNFR1 and TNFR2 signaling in the heart has been demonstrated [45]. TNFR1 plays a pro-inflammatory and apoptotic role after an activation of the NF-kB and JNK pathways. However, TNFR2 induces an anti-inflammatory effect to counter the effects of TNFR1 on tissue repair. In the clinic, TNF-α levels are known to increase and TNF-α receptors are known to significantly decrease in patients with chronic heart failure [46]. Therefore, variations in TNFR1 and TNFR2 signaling and regulation during cardiac disorders remain unclear.

In the present study, etanercept was observed to ameliorate cardia fibrosis in HFD-fed rats mainly through a reduction in STAT3 activation. STAT3 plays a major role in fibrosis [47] and is known to act both independently and in conjunction with other signaling networks such as TGF-β1 signaling [48]. The essential role of JAK/STAT3 signaling in the pathogenesis of fibrosis has been demonstrated in a recent review [49]. TNFα initiates the activation of JAK/STAT3, which is dependent on TNFR2 but not TNFR1, at a low dose [50]. In ischemic hearts, lower doses of TNFα reduce infarct size, while higher doses of TNFα increase infarct size [51]. In the present study, HFD-fed rats exhibited an increased TNFα in the tissues [14]. Therefore, high concentrations of TNFα in HFD-fed rats may contribute to cardiac dysfunction, including hypertrophy, fibrosis, and apoptosis, most likely via TNFR1 activation [51]. The cardiac signaling system downstream of TNFR1 has been well studied [52]. Additionally, the direct activation of p38 by TNFα may lead to IL-6 production [53]. Cytokines, such as IL-1β, TNFα, and IL-6, are elevated in chronic inflammation, such as rheumatoid arthritis (RA), through the direct or indirect activation of STAT3, while STAT3 activation may further induce IL-6 and its family cytokines [54]. Therefore, the IL-6/JAK/STAT3 signaling pathway is involved in TNFα-associated inflammation. Moreover, the role of nuclear factor-κB (NFkB) in the regulation of cardiac fibrosis has also been described [55], and it is known to be elevated after TNFR1 activation [17]. NFkB signaling occurs through a canonical or classical pathway, or a noncanonical or alternative pathway [56]. TNFα is well- known to activate canonical NFkB signaling [56]. NFkB signaling is also activated by the IL-6 cytokine [57]. The chronic activation of NFkB signaling results in prolonged inflammation, inducing increased apoptotic cell death and progression toward heart failure [58] through the enhanced secretion of cytokines, including TNFα, IL-1, and IL-6 [59]. Therefore, the IL-6/JAK/STAT3 signaling pathway is also activated in NFkB-induced cardiac injury. Overall, the cardioprotective and cardiotoxic roles of NFkB signaling in the heart are considered to depend on acute and chronic responses [59].

Anti-TNF therapy is widely used in clinics [60] and the agents used, including etanercept, are known to interrupt both receptors of TNFα. Stimulating TNFR1 in cardiac myocytes is the major influence of TNFα, and the cytotoxic effects of TNFα are also mediated through TNFR1 [50]. In the present study, etanercept inhibited the increase in inflammatory cytokines, including TNFα, IL-1, and IL-6, in addition to NFkB in HFD-fed rats. Additionally, etanercept is found to ameliorate cardiac fibrosis in HFD-fed rats mainly through the reduction in STAT3 activation which plays a major role in fibrosis [47]. The IL-6 linked STAT3 activation seems to be the major target of etanercept. In H9c2 cells, the inhibition of STAT3 activation by etanercept was less effective than that by static, which may directly interrupt STAT3 [61]. Therefore, the inhibition of IL-6 by etanercept through reduction of TNFα or TNFR1 seems to be associated with the decrease in IL-6 linked STAT3 activation in HFD-fed rats. However, this hypothesis needs to investigate in detail using the STAT3-null mice in the future. Additionally, TNFR2- coupled JAK/STAT3 signaling has been identified in cancer cells [62] and it is also activated in brain neuroinflammation, including the neuroinflammation in Alzheimer’s disease [63]. Therefore, specific inhibitors of TNFR1 or TNFR2 are urgently required, not only for bench research but also for clinical applications.

## 4. Materials and Methods 

### 4.1. Materials

Etanercept (Enbrel) was the product of Wyeth Europa (Maidenhead, UK). Stattic and palmitic acid (palmitate, PA) were purchased from Sigma-Aldrich (St. Louis, MO, USA); stock solutions of 5 mM PA/10% BSA were prepared and stored at −20°C. The stock solution was heated to room temperature prior to use.

### 4.2. Animal Model

Male Sprague-Dawley (SD) rats weighing 250 to 270 g were purchased from the National Laboratory Animal Center (Taipei, Taiwan). The animal experiments were approved (105110327) by the Institutional Animal Ethics Committee of Chi-Mei Medical Center. The animal experiments were performed in accordance with the Guide for the Care and Use of Laboratory Animals of the National Institutes of Health, and the guidelines of the Animal Welfare Act. 

The 8-week-old rats were divided into three groups (*n*=6 each): (i) rats fed normal chow as normal control; (ii) HFD-fed rats treated with vehicle as a model control; and (iii) HFD-fed rats treated with etanercept. The HFD-fed rats were established by the free intake of a diet containing 60% (wt/wt) fat (58Y1; TestDiet, Richmond, IN, USA) for 16 weeks as described previously [23]. Etanercept (Enbrel, Wyeth Europa, Maidenhead, UK) was subcutaneously injected at 0.8 mg/kg/week twice weekly during the last 8 weeks of HFD feeding [37]. Vehicle treatment was performed in the same manner by injecting sterile water, the solvent for etanercept. After the end of treatment, the body weight of each rat was measured. Then, blood samples were collected from femoral artery after 12 h fasting for analysis. After measurement of cardiac performance, the heart was surgically extirpated, washed in ice-cold saline, dried, and immediately weighed. The heart tissues were stored at −20 °C until further analysis. The rats were used under anesthesia (2% isoflurane) to minimize suffering. The plasma was prepared by centrifugation at 2000 g for 15 min at 4 °C and then stored at −80 °C until further analysis. 

Plasma glucose level was measured, following a previous method [64], by the glucose oxidase method using an analyzer (Quik-Lab, Ames; Miles Inc., Elkhart, IN, USA). 

### 4.3. Analysis of Plasma Lipids

The lipid profiles, including triglycerides, were determined using commercial kits (Cayman Chemical, Ann Arbor, MI, USA). The total cholesterol, HDL were determined using commercial kits (abcam, Cambridge, MA, USA), according to the supplier’s instructions. LDL was estimated by the formula: LDL = total cholesterol − HDL − triglyceride/5.

### 4.4. Measurement of Blood Biomarkers

The plasma levels of the pro-inflammatory cytokines, TNFα, IL-1β, and the anti-inflammatory factor, IL-6, were estimated using ELISA kits purchased from Sigma Aldrich (St. Louis, MO, USA). Plasma ALT and aspartate AST levels were measured using an autoanalyzer and a commercially kit (Roche, Germany). 

### 4.5. Cardiac Performance Measurements

Measurements of cardiac performance were carried out using our previously described method [65]. All the rats were maintained at 37.5 °C during the experiments. In brief, pressure transducer catheters were inserted to obtain the right ventricular, aortic, mean blood, and left ventricular pressures. An external pacing computer (iWorx Systems, Inc., Dover, NH, USA) connected to transducers was used to acquire the hemodynamic signals. Then, we compared the main indices: namely, heart rate (HR), LV systolic pressure (LVSP), LV end-diastolic pressure (LVEDP), and the maximal and minimal rates of LV pressure change (± dp/dtmax), as described in our previous report [65]. 

### 4.6. Morphological Evaluation

Whole hearts from the sacrificed rats were used to fix with 4% paraformaldehyde at 4 °C for 24 h and embedded in paraffin. Five-micron sections were obtained, deparaffinized, and rehydrated. The sections were then subjected to the hematoxylin and eosin (H&E) staining. Each section was examined for evidence of mononuclear and polymorphic cell infiltration, necrosis and mineralization. In addition, the collagen content in the heart tissues was estimated by Masson trichrome staining. The ratio of the collagen positive area to the total area was calculated (%) through a computer imaging system [66]. 

### 4.7. Cell Culture

H9c2 cells (BCRC No. 60096) were cultured as previously described [67]. Briefly, the H9c2 cells were maintained in Dulbecco’s modified Eagle’s medium (DMEM, pH 7.2; Gibco-BRL Life Technologies, Gaithersburg, MD, USA) supplemented with 10% fetal bovine serum. The cells were plated at 6000 cells/cm^2^ and were allowed to proliferate in the medium. The medium was replaced on the second day. Then, the cells were used for the subsequent experiments because H9c2 cells showed similar responses as primary rat neonatal cardiomyocytes [67]. The cells were pretreated with the test agent at the indicated concentration for 1 h, followed by incubation with palmitate (250 μM) for 48 h [68]. Following the treatments, the cells were collected for analysis. 

### 4.8. Real-Time Quantitative PCR

In brief, total RNA was extracted from the tissue or cell lysates with TRIzol reagent (Carlsbad, CA, USA) [69]. The total RNA was reverse-transcribed into cDNA with random hexamer primers (Roche Diagnostics GmbH, Mannheim, Germany). The PCR experiments were performed using a LightCycler (Roche Diagnostics GmbH, Mannheim, Germany). The concentration of each product was calculated based on a corresponding standard curve. The relative gene expression was subsequently indicated as the ratio of the target gene level to that of β-actin. The primers for each factor are shown as follows: 

MCP-1

F: 5′-TCTCTTCCTCCACCACTATGCA-3′

R: 5′-GGCTGAGACAGCACGTGGAT-3′

NFkB

F: 5′-TGAGTCCCGCCCCTTCTAA-3′

R: 5′-TGATGGTCCCCCCAGAGA-3′

TGF-β1

F: 5′-AGGTCACCCGCGTGCTAAT-3′

R: 5′-TCAACCACTGCCGCACAACT-3′

JAK2

F: 5′-TTTGGATCCCTGGATACATACCTGA-3′

R: 5′-TGGCACACACATTCCCATGA-3′

β-actin 

F: 5′-CTAAGGCCAACCGTGAAAAG-3′;

R: 5′-GCCTGGATGGCTACGTACA-3′

### 4.9. Western Blotting Analysis

The ice-cold radio-immuno-precipitation assay (RIPA) buffer was used to extract the proteins from heart homogenates or cell lysates [66]. The target antigens from the protein extracts were characterized using the primary antibodies specific for p-STAT3, STAT3, CCN2, fibronectin, or β-actin (Abcam, Cambridge, MA, USA). The bound primary antibodies were then hybridized to horseradish peroxidase-conjugated goat anti-rabbit or anti-mouse IgGs (Calbiochem, San Diego, CA, USA). The optical densities of each band indicating p-STAT3 (88 kDa), STAT3 (88 kDa), CCN2 (38 kDa), fibronectin (270 kDa), or β-actin (43 kDa) were quantified using our previously described method [66].

### 4.10. Statistical Analysis

The results are shown as the mean± SEM of each group. The significance of the variations between groups was evaluated by a Student’s *t*-test for unpaired data or Dunnett’s t-test for multiple comparisons preceded by one-way analysis of variance (ANOVA). *p* < 0.05 was considered statistically significant. 

## 5. Conclusions

We demonstrated a novel view that etanercept is effective in ameliorating cardiac fibrosis in rats with diet-induced obesity through the inhibition of TNFα and/or its receptor activation. The etanercept treatment of HFD-fed rat reduced the body weight gain and prevented elevations of the other biochemical parameters included triglyceride, LDL, total cholesterol, AST, and ALT. Then, etanercept may decrease the STAT3 activation to reduce the expression of fibrosis-related genes, including fibronectin and CTGF, which are increased in HFD-fed rats. Therefore, anti-TNF therapy is a possible therapeutic option for cardiac fibrosis that could be developed after careful clinical trials.

## Figures and Tables

**Table 1:** Effects of etanercept on the changes in body weight, heart weight, blood glucose and lipid levels, and other parameters in high fat diet (HFD)-fed rats.

Parameters	Normal Rat	HFD-fed Rat	+Etanercept
	(i)	(ii)	(iii)
Body weight(g)	447.33 ± 16.49	549.50 ± 19.97 *	463.17 ± 17.09
Heart weight(g)	2.28 ± 0.08	2.74 ± 0.15	2.36 ± 0.13
Blood glucose(mg/dL)	116.50 ± 5.58	157.83 ± 7.05 *	139.00 ± 4.15 ^#^
Total Cholesterol(mg/dL)	76.92 ± 4.73	190.14 ± 3.41 *	169.24 ± 6.59 ^#^
Triglyceride(mg/dL)	86.86 ± 2.13	236.71 ± 4.53 *	212.74 ± 4.86 ^#^
HDL (mg/dL)	27.17 ± 1.74	20.46 ± 0.76 *	25.20 ± 1.07 ^#^
LDL (mg/dL)	32.38 ± 4.59	122.34 ± 4.51 *	101.50 ± 5.56 ^#^
AST (U/L)	114.00 ± 6.20	226.50 ± 5.60 *	168.00 ± 5.60 ^#^
ALT (U/L)	44.20 ± 3.50	132.70 ± 3.50 *	109.30 ± 7.70 ^#^

The rats were divided into three groups: (**i**) normal chow-fed control rats; (**ii**) vehicle-treated HFD-fed rats; and (**iii**) etanercept-treated HFD-fed rats. The HFD-fed rats were given a commercial diet for 16 weeks. Etanercept (Enbrel) was given 0.8 mg/kg/week twice weekly via subcutaneous injection during the last 8 weeks of HFD feeding. Vehicle treatment was given in the same manner with sterile water, the solvent for etanercept. After the treatment, blood samples were collected from femoral artery after 12 h of fasting for analysis. The data are shown as the mean ± standard deviation (*n* = 6 per group). * *p* < 0.05 vs. control group, # *p* < 0.05 vs. vehicle-treated group, Student’s *t*-test for unpaired data. HFD, high fat diet; HDL, high density lipoprotein; LDL, low density lipoprotein; AST, aspartate aminotransferase; ALT, alanine aminotransferase.

## Data Availability

The data is confidentiality.
